# Factors Associated With Anterior Chamber Depth Changes in Primary Angle-Closure Suspects Over 5-Year Follow-Up

**DOI:** 10.1167/tvst.15.10.37

**Published:** 2026-10-23

**Authors:** Fei Xiang, Katherine Han, Mengyang Li, Gui-shuang Ying, Xiaohua Pan, Hui Feng, Caixia Li, Liqin Song, Yi Wang, Junying Ren, Zehua Wang, Zhi Li, Na Jiang, Ying Han, Shuning Li

**Affiliations:** 1 Beijing Tongren Eye Center, Beijing Tongren Hospital, Beijing Ophthalmology & Visual Sciences Key Laboratory, Capital Medical University, Beijing, China; 2 Albert Einstein College of Medicine, Bronx, NY, USA; 3 Center for Preventive Ophthalmology and Biostatistics, Department of Ophthalmology, Perelman School of Medicine at the University of Pennsylvania, Philadelphia, PA, USA; 4 Shenzhen Eye Hospital, Jinan University, Shenzhen Eye Institute, Shenzhen, China; 5 Shaanxi Eye Hospital, Xi'an People's Hospital (Xi'an Fourth Hospital), Xi'an, China; 6 Chen Zhou AIER Eye Hospital, Hunan, China; 7 Department of Ophthalmology, University of California, San Francisco, School of Medicine, San Francisco, CA, USA

**Keywords:** anterior chamber parameters, primary angle-closure suspect, glaucoma

## Abstract

**Purpose:**

To evaluate longitudinal changes in anterior chamber depth (ACD) in primary angle-closure suspects (PACS) over 5-year follow-up and identify factors associated with these changes.

**Methods:**

Participants aged 50 years or older were enrolled from Daxing District, Beijing, China. Anterior chamber parameters were assessed at baseline and at 5-year follow-up using anterior segment optical coherence tomography and Lenstar 900. Baseline demographics, ocular characteristics, and longitudinal changes in anterior chamber parameters were compared between groups. Univariate and multivariate linear regression analyses were performed to identify factors associated with changes in ACD.

**Results:**

A total of 101 eyes with PACS and 136 control eyes without PACS (NPACS) were analyzed. At 5-year follow-up, both groups showed significant decreases in ACD and significant increases in lens thickness, lens thickness/axial length factor (LAF), and lens vault (LV) compared with baseline (all *P* < 0.001). In multivariate subgroup analyses stratified by baseline age (group A: 50–60 years, *n* = 128; group B: >60 years, *n* = 109), ACD shallowing in group A was associated with hypermetropia, greater follow-up to baseline change in LAF, and greater follow-up to baseline change in LV (all *P* < 0.001). In group B, ACD shallowing was associated with diagnosis of PACS (*P* = 0.027) and greater follow-up to baseline change in LV (*P* < 0.001).

**Conclusions:**

ACD decreased over 5 years in participants with both PACS and NPACS. ACD shallowing was associated with both lens thickening and increased LV in relatively younger participants, whereas it was associated mainly with increased LV in relatively older participants.

**Translational Relevance:**

These findings suggest that lens enlargement and anterior lens displacement may contribute differently to ACD shallowing across age groups, which may aid in the interpretation of longitudinal anterior segment changes in PACS.

## Introduction

Primary angle-closure suspect (PACS) is defined as the early stage in the spectrum of primary angle-closure disease (PACD), according to the International Society of Geographic and Epidemiologic Ophthalmology classification.^1^ PACS is characterized by a shallow anterior chamber, a narrow chamber angle, a relatively thick lens, and so on.^2^ In China, more than 28 million people are estimated to have PACS.^3^ However, longitudinal data from the Zhongshan Angle Closure Prevention Trial demonstrated that only a small proportion of PACS eyes progressed to primary angle closure (PAC) or primary angle-closure glaucoma (PACG), which are the two later stages of PACD. Identifying which individuals with PACS are at high risk of progression remains a major clinical challenge.

Shallow anterior chamber depth is a key risk factor for PACG. The shallower the anterior chamber, the greater the risk of disease progression.^4^ Previous studies have shown that anterior chamber depth (ACD) decreased with aging,^5^ which may potentially explain the increasing incidence of PACG in older populations. However, few longitudinal studies have observed and evaluated age-related changes in ACD over time. Therefore, we aim to compare 5-year changes in ACD between PACS and non-PACS (NPACS) populations and to explore factors associated with these changes.

## Materials and Methods

### Ethics Statement

The study was approved by the Ethics Committee of Clinical Research of Beijing Tongren Hospital (approval number: TREC2022-KY022) and adhered to the tenets of the Declaration of Helsinki. Written and verbal informed consent was obtained from all participants.

### Study Design and Population

This was a community-based, observational, longitudinal study of residents aged 50 years or older from Zhangziying Town, Daxing District, Beijing, China. Participants were identified from local resident records maintained by the local government health service center and were required to have resided in the town for at least 6 months. The baseline examination was conducted in October 2018, with a 5-year follow-up examination in July 2023. Participants with PACS who had readable anterior segment optical coherence tomography (AS-OCT) images at both baseline and the 5-year follow-up were included. PACS was defined by the following inclusion criteria: pigmented trabecular meshwork invisible for 180° or more in the primary position under static gonioscopy, absence of peripheral anterior synechiae, intraocular pressure (IOP) ≤21 mm Hg, and absence of glaucomatous optic neuropathy (GON). Gonioscopy was performed by two experienced glaucoma specialists (SL and ML). The exclusion criteria included the presence of suspectable GON (defined as vertical cup/disc ratio [VCDR] ≥0.7 or an intereye difference in VCDR >0.2), optic disc hemorrhages, optic nerve rim loss or notch, retinal nerve fiber layer defects on spectral-domain ocular coherence tomography, prior iris laser treatment (laser iridotomy and laser iridoplasty) or intraocular surgery, history of intraocular inflammation, trauma, congenital malformation, secondary angle closure, or prior episode of acute PAC in either eye. In addition, eyes that underwent iris laser treatment or intraocular surgery during follow-up were also excluded from the longitudinal analysis. Participants with poor-quality AS-OCT images or missing data were also excluded. Patients with NPACS had normal eye examination results and open-angle structures on gonioscopy and were subject to the same exclusion criteria. For each participant, only eyes that met all inclusion and exclusion criteria were included. If both eyes were eligible, the right eye was selected.

### Clinical Examinations

All participants underwent a basic physical examination and a comprehensive ocular examination. Age, sex, heart rate, and random blood glucose were measured during the physical examination. Eye examinations included visual acuity, objective refraction (Topcon Corporation, Tokyo, Japan), noncontact tonometry (Topcon Corporation), slit-lamp examination (Haag Streit, Bern, Switzerland), gonioscopy (Ocular, Bellevue, WA, USA), fundus photography (Kowa Nonmyd WX, Tokyo, Japan), ocular biometry measurement (Lenstar LS900; Haag Streit), and AS-OCT scans (CASIA SS-1000; Tomey Corporation, Nagoya, Japan). Data collected included best-corrected visual acuity (BCVA), IOP, spherical equivalence, central corneal thickness (CCT), lens thickness (LT), axial length (AL), ACD, lens vault (LV), and iris area (Iarea). The IOP was measured three times by Goldmann applanation, and the mean value was used for statistical analysis.

The design principle of the Lenstar 900 is optical low-coherence reflectometry, utilizing an 820-nm superluminescent diode as its light source to achieve high resolution and accuracy. Anterior chamber parameters, including CCT, ACD, LT, and AL, were measured by the Lenstar 900. ACD was defined as the distance from the posterior surface of the cornea to the anterior surface of the crystalline lens. Lens thickness/axial length factor (LT/AL factor, LAF) was the ratio of LT to AL multiplied by 10, which reflects the lens's relative proportion within the eye.

Anterior segment images were obtained using AS-OCT in a dark room (approximately 10 lux). Following the administration of topical anesthetic, an eye speculum was used to hold the eyelids in place to ensure clear visualization of limbal structures. Image analysis was performed using the built-in “2D Analysis” mode of the AS-OCT software. After manual identification of the scleral spur on both sides, measurements of LV and Iarea on each side were automatically reported. LV was defined as the straight-line distance from the anterior surface of the crystalline lens to the midpoint of the straight line connecting the scleral spurs. Iarea was calculated as the average of iris areas on both sides. All images were labeled by two experienced and well-trained examiners (FX and ZL), who were masked to the categorization of participants into the PACS or NPACS groups.

To evaluate interobserver repeatability, 10 randomly selected anterior segment images—not included in either PACS or NPACS groups—were independently assessed by the two examiners (FX and ZL). Intraclass correlation coefficients (ICCs) were calculated for LV and Iarea. The ICCs for LV and larea were 0.88 (95% confidence interval [CI], 0.32–0.97) and 0.92 (95% CI, 0.51–0.98), respectively, indicating outstanding measurement consistency.

### Statistical Analysis

The normality of the distribution of quantitative measures was evaluated using the one-sample Kolmogorov–Smirnov test. Measures were summarized using mean ± standard deviation (SD) for those following a normal distribution and median and quartiles for those not normally distributed. For exploratory age-stratified analyses, participants were further divided into two subgroups based on baseline age: 50 to 60 years and >60 years. The cutoff of 60 years was selected to approximate the median baseline age of the study population and to ensure relatively balanced subgroups. Comparisons between NPACS and PACS, as well as between the two age groups (50–60 years vs. >60 years), were conducted using the two-sample *t*-test (for continuous measures with normal distribution) or the Mann–Whitney *U* test (for continuous measures with nonnormal distribution) and the χ^2^ test for categorical measures. Adjustments for pupil diameter were performed when comparing iris areas between two groups. Changes from baseline (△) at the 5-year follow-up for each anterior chamber parameter were calculated by subtracting the baseline measurements from the measurements taken at 5 years. Paired *t*-tests and Wilcoxon signed-rank tests were used to assess whether changes in parameters from baseline to the 5-year follow-up were statistically significant. An initial significance level of two-sided *P* < 0.05 was used, with Bonferroni correction applied as appropriate to adjust for multiple comparisons. Univariate and multivariate linear regression analyses were employed to identify factors associated with changes in anterior chamber parameters. Variables with *P* < 0.10 in the univariate analyses were entered into the initial multivariable linear regression model. Backward stepwise selection was then performed by sequentially removing the least significant variable at each step until only variables with *P* < 0.05 remained. Multicollinearity among candidate variables was assessed using the variance inflation factor prior to multivariable analysis. All statistical analyses were performed using the Statistical Package for Social Sciences (SPSS) for Windows, version 26.0 (IBM Corporation, Armonk, NY, USA).

## Results

Of the 380 participants who received examinations at baseline and at the 5-year follow-up, 237 eyes from the 237 participants met the eligibility criteria and were included in the study (Fig.). Of these, 136 eyes were diagnosed with NPACS and 101 eyes with PACS. Baseline demographic and ocular characteristics comparison between PACS and NPACS groups is summarized in Table 1. Compared with NPACS eyes, eyes with PACS exhibited significantly more hypermetropia (*P* < 0.001), thicker lens (*P* < 0.001), shorter AL (*P* < 0.001), larger LAF (*P* < 0.001), shallower ACD (*P* < 0.001), and larger LV (*P* < 0.001).

**Figure.**
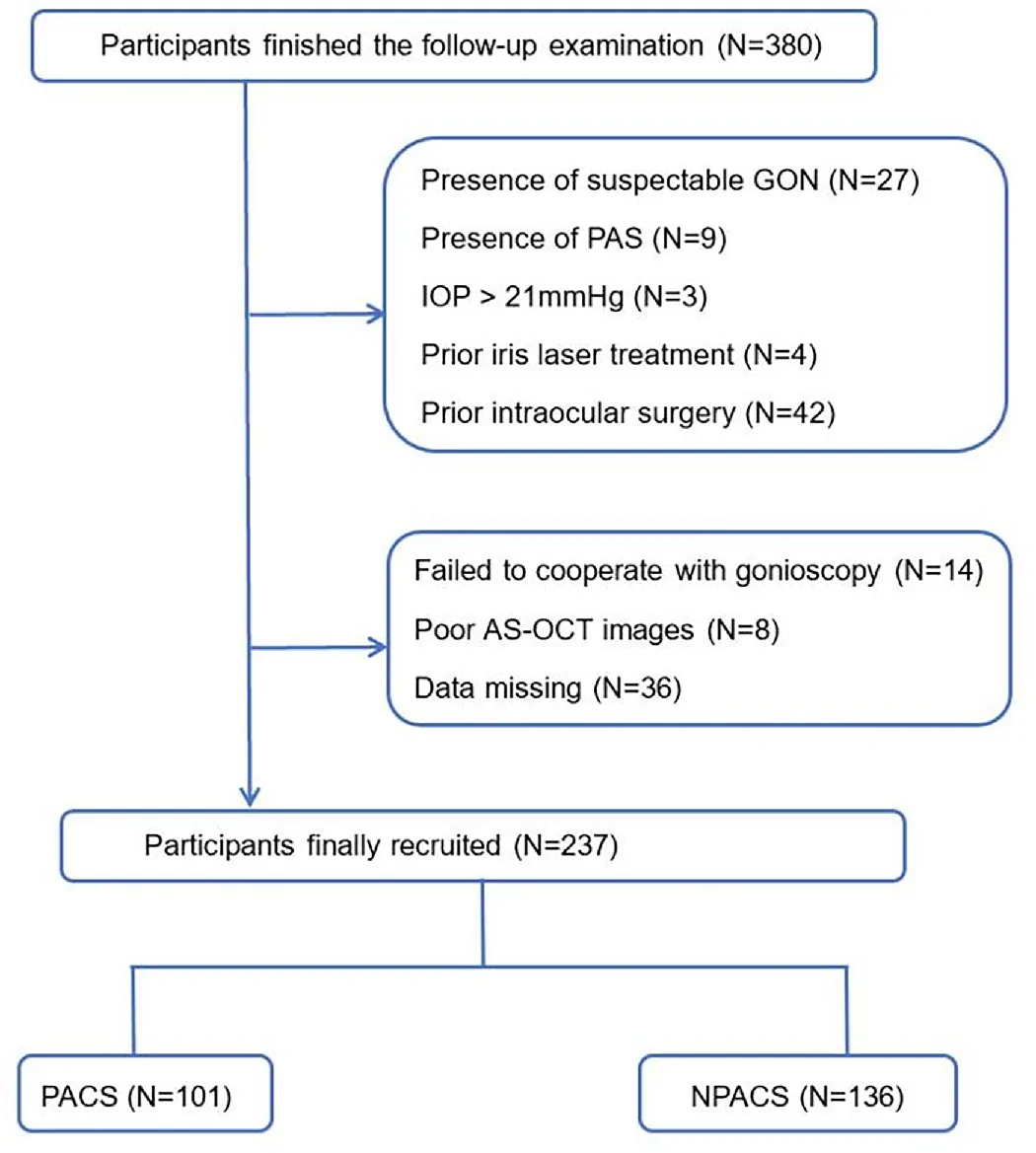
Participants’ enrollment flowchart. PAS, peripheral anterior synechiae.

**Table 1.** Comparison of Baseline Demographic and Ocular Characteristics Between PACS and NPACS Participants

Parameter	NPACS (*n* = 136)	PACS (*n* = 101)	*P* Value
Demographic characteristics			
Age (IQR), years	59.00 (56.00, 65.00)	61.00 (56.50, 65.00)	0.25^a^
Gender (male/female)	37/99	21/80	0.29^b^
Random blood glucose (IQR), mmol/L	7.30 (6.00, 8.80)	7.55 (5.80, 9.88)	0.57^a^
Heart rate (mean ± SD), bpm	84.25 ± 12.61	82.51 ± 11.54	0.28^c^
Ocular characteristics			
BCVA (IQR), decimal visual acuity	1.00 (0.80, 1.00)	1.00 (0.80, 1.00)	0.78^a^
IOP (IQR), mm Hg	17.00 (15.00, 18.00)	16.00 (15.00, 18.00)	0.17^a^
SE (IQR), diopters	0.25 (−0.38, 0.88)	0.88 (0.25, 1.63)	**<0.001** ^a^ [Table-fn tb1fn3]
CCT (mean ± SD), mm	531.57 ± 31.83	529.36 ± 29.57	0.59^c^
LT (mean ± SD), mm	4.45 ± 0.31	4.67 ± 0.31	**<0.001** ^c^
AL (mean ± SD), mm	23.06 ± 0.79	22.52 ± 0.75	**<0.001** ^c^
LAF (mean ± SD)	1.93 ± 0.15	2.07 ± 0.15	**<0.001** ^c^ [Table-fn tb1fn1]
ACD (IQR), mm	2.64 (2.49, 2.76)	2.29 (2.11, 2.43)	**<0.001** ^a^
LV (mean ± SD), mm	0.38 ± 0.24	0.65 ± 0.20	**<0.001** ^c^ [Table-fn tb1fn1]
Iarea (mean ± SD), mm^2^	1.42 ± 0.17	1.42 ± 0.18	0.59^d^

*P* values with statistical significance are shown in boldface; *P* < 0.05 was considered statistically significant. IQR, interquartile range; SD, standard deviation; SE, spherical equivalent.

a Mann–Whitney test.

b χ^2^ test.

c Two-sample *t*-test.

d *P* value with adjustment for pupil diameter.

Comparison of anterior chamber parameters at baseline and 5-year follow-up in PACS and NPACS participants is presented in Table 2. Bonferroni correction was applied, adjusting the significance threshold to α = 0.007 (0.05/7). Both PACS and NPACS eyes showed a significant decrease in ACD (*P* < 0.001), an increase in LT (*P* < 0.001), an increase in LAF (*P* < 0.001), and an enlargement of LV (*P* < 0.001) over the 5-year follow-up period.

**Table 2.** Comparison of Anterior Chamber Parameters at Baseline and 5-Year Follow-Up in PACS and NPACS Participants

	NPACS (*n* = 136)			PACS (*n* = 101)		
	Baseline	Follow-up	*P* Value^a^	Baseline	Follow-up	*P* Value^a^
CCT, mm	531.57 ± 31.83	530.09 ± 31.48	0.03^b^	529.36 ± 29.57	529.29 ± 29.22	0.91^c^
ACD, mm	2.64 (2.49, 2.76)	2.58 ± 0.28	**<0.001** ^c^	2.27 ± 0.24	2.21 ± 0.24	**<0.001** ^b^
LT, mm	4.45 ± 0.31	4.61 ± 0.32	**<0.001** ^b^	4.67 ± 0.31	4.83 ± 0.30	**<0.001** ^b^
AL, mm	23.06 ± 0.79	23.08 ± 0.80	0.03^b^	22.52 ± 0.75	22.52 ± 0.75	0.70^b^
LAF	1.93 ± 0.15	2.00 ± 0.15	**<0.001** ^b^	2.05 ± 0.13	2.13 ± 0.13	**<0.001** ^b^
LV, mm	0.38 ± 0.24	0.43 ± 0.24	**<0.001** ^c^	0.66 ± 0.20	0.70 ± 0.21	**<0.001** ^b^
Iarea, mm^2^	1.42 ± 0.17	1.34 ± 0.19	0.92^d^	1.42 ± 0.18	1.34 ± 0.17	0.11^d^

Measures are reported as mean ± standard deviation for those following a normal distribution and median (interquartile range) for skewed data. Values with statistical significance are shown in boldface.

a Bonferroni correction applied for multiple comparisons; significance threshold adjusted to α = 0.007 (0.05/7).

b Wilcoxon signed-rank test.

c Paired-sample *t*-test.

d *P* value with adjustment for pupil diameter.

In the exploratory age-stratified analysis, participants were further divided into two subgroups according to baseline age: group A included participants aged 50 to 60 years (*n* = 128), and group B included those older than 60 years (*n* = 109). Compared to group A, group B had significantly lower BCVA (*P* < 0.001), more hypermetropia (*P* < 0.001), thicker lens (*P* < 0.001), and larger LAF (*P* < 0.001). Detailed subgroup data are presented in Supplementary Table S1.

When comparing 5-year changes from baseline between PACS and NPACS eyes across all participants, no parameter showed a significant difference between the PACS and NPACS groups (Supplementary Table S2). In subgroup analysis (Table 3), a greater reduction in ACD was observed in PACS eyes compared with NPACS eyes among participants in group B (aged >60 years) (*P* = 0.04). However, this difference did not remain statistically significant after Bonferroni correction for multiple comparisons (adjusted significance threshold α = 0.007). Despite this, the initial significance and the low adjusted *P* value suggest potential clinical relevance that merits further investigation. Given the essential role of ACD in the pathophysiology and progression of PACD, we considered it meaningful to further explore factors associated with ACD reduction within the two age subgroups.

**Table 3.** Five-Year Changes of Anterior Chamber Parameters Between PACS and NPACS Participants, Stratified by Baseline Age Group

	Group A: Baseline Age 50–60 Years (*n* = 128)			Group B: Baseline Age >60 Years (*n* = 109)		
Variable	NPACS (*n* = 78)	PACS (*n* = 50)	*P* Value^a^	NPACS (*n* = 58)	PACS (*n* = 51)	*P* Value^a^
△CCT, mm	−1.00 (−5.00, −3.00)	0.00 (−5.00, −4.00)	0.38^b^	−1.00 ± 6.18	0.35 ± 6.91	0.28^c^
△ACD, mm	−0.06 ± 0.08	−0.07±0.10	0.56^c^	−0.03 ± 0.07	−0.05 ± 0.06	0.04^c^
△LT, mm	0.14 (0.08, 0.22)	0.15 (0.09, 0.23)	0.57^b^	0.12 (0.06, 0.21)	0.10 (0.07, 0.17)	0.71^b^
△AL, mm	0.01 (−0.03, 0.04)	−0.01 (−0.04, 0.02)	0.07^b^	0.01 (−0.02, 0.06)	0.01 (−0.01, 0.04)	0.99^b^
△LAF	0.06 (0.03, 0.09)	0.07 (0.04, 0.10)	0.37^b^	0.05 (0.02, 0.09)	0.04 (0.03, 0.07)	0.84^b^
△LV, mm	0.06 ± 0.128	0.06 ± 0.119	0.84^c^	0.04 (−0.03, 0.10)	0.03 (−0.03, 0.07)	0.49^b^
△Iarea, mm^2^	0.09 (0.00, 0.16)	0.08 (0.02, 0.13)	0.80^d^	0.12 ± 0.12	0.08 ± 0.10	0.09^d^

Measures are reported as mean ± standard deviation for those following a normal distribution and median (interquartile range) for skewed data. Values with statistical significance are shown in boldface. Δ, follow-up to baseline changes (follow-up minus baseline).

a Bonferroni correction applied for multiple comparisons; significance threshold adjusted to α = 0.007 (0.05/7).

b Mann–Whitney test.

c Two-sample *t*-test.

d *P* value with adjustment for pupil diameter.

Univariate and multivariate linear analyses were performed for participants in group A (aged 50–60 years; Table 4) and group B (aged >60 years; Table 5). In group A, three factors were significantly associated with ACD shallowing in the multivariate analysis: hypermetropia (β = 0.278, *P* < 0.001), larger follow-up to baseline change in LAF (β = −0.252, *P* < 0.001), and larger follow-up to baseline change in LV (β = −0.496, *P* < 0.001) (Table 4). In contrast, in older-aged group B, two factors were significantly associated with ACD shallowing: a diagnosis of PACS (β = −0.196, *P* = 0.027) and greater follow-up to baseline change in LV (β = −0.341, *P* < 0.001) (Table 5).

**Table 4.** Univariate and Multivariate Regression Analyses for Factors Associated With ACD Change in Group A: Baseline Aged 50 to 60 Years (*n* = 128)

	Univariate Analysis		Multivariate Analysis		
Variable	Standardized Regression Coefficient β	*P* Value	Standardized Regression Coefficient β	*P* Value	Variance Inflation Factor
Baseline parameter					
Age	0.205	**0.02**			
Gender	0.058	0.52			
Random blood glucose	−0.219	**0.01**			
Heart rate	−0.090	0.32			
BCVA	−0.064	0.47			
IOP	0.040	0.66			
SE	0.195	**0.03**	0.278	**<0.001**	1.060
Diagnosis of PACS	−0.053	0.56			
Changes in anterior chamber parameters					
△CCT	0.101	0.26			
△LT	−0.335	**<0.001**			
△AL	0.280	**0.001**			
△LAF	−0.364	**<0.001**	−0.252	**<0.001**	1.063
△LV	−0.491	**<0.001**	−0.496	**<0.001**	1.049
△Iarea	−0.063	0.51^a^			

*P* values with statistical significance are shown in boldface; *P* < 0.05 was considered statistically significant. Δ, follow-up to baseline changes (follow-up minus baseline).

a *P* value with adjustment for pupil diameter.

**Table 5.** Univariate and Multivariate Regression Analyses for Factors Associated With ACD Change in Group B: Baseline Aged >60 Years (*n* = 109)

	Univariate Analysis		Multivariate Analysis		
Variable	Standardized Coefficient β	*P* Value	Standardized Coefficient β	*P* Value	Variance Inflation Factor
Baseline parameter					
Age	0.141	0.14			
Gender	−0.007	0.94			
Random blood glucose	0.079	0.42			
Heart rate	0.054	0.58			
BCVA	−0.033	0.73			
IOP	−0.049	0.61			
SE	−0.042	0.66			
Diagnosis of PACS	−0.197	**0.040**	−0.196	**0.027**	1.001
Changes in anterior chamber parameters					
△CCT	−0.049	0.62			
△LT	−0.215	**0.025**			
△AL	0.288	**0.002**			
△LAF	−0.225	**0.019**	−0.171	0.055	1.022
△LV	−0.364	**<0.001**	−0.341	**<0.001**	1.021
△Iarea	0.136	0.48^a^			

*P* values with statistical significance are shown in boldface; *P* < 0.05 was considered statistically significant. Δ, follow-up to baseline changes (follow-up minus baseline).

a *P* value with adjustment for pupil diameter.

## Discussion

To our knowledge, this is the first longitudinal study to characterize changes in ACD in eyes with and without PACS. Both groups showed a significant decrease in ACD, an increase in LT, an increase in LV, and an enlargement in LAF. In the 50 to 60 years age group, hypermetropia and greater follow-up to baseline change in both LAF and LV were significant associations with ACD reduction. In contrast, in the over 60 years age group, a diagnosis of PACS and a greater follow-up to baseline change in LV were significantly associated with greater ACD shallowing.

Few cohort studies have provided longitudinal data in PACS populations, as prior studies of age-related anterior segment changes in PACD have been largely cross-sectional.^6^^,^^7^ Other studies have focused on the changes in anterior segment morphology after laser iridotomy.^8^^–^^10^ Longitudinal data in eyes with open angles have been reported by He et al.,^11^ who observed a significant decrease in mean ACD and an increase in LV over 4 years in a cohort of Singaporean participants. This result supports the notion that ACD decreases with aging. This is also consistent with epidemiologic studies showing that PACG prevalence increases with age.^12^^,^^13^ Given that ACD is a well-established anatomic risk factor for PACD,^14^ progressive shallowing may contribute to disease development and progression. In line with this, Yuan et al.^15^ reported that shallower central and limbal ACDs may serve as baseline predictors for disease progression in PACS eyes.

The mechanisms underlying age-related decreases in ACD remain incompletely understood. While lens thickening with aging has been proposed as a primary contributor,^16^ the relative roles of lens enlargement and anterior displacement are still not well defined. In our study, LT, LV, and LAF, parameters reflecting the relative position and size of the lens within the globe, were compared between PACS and NPACS populations over time. Both groups demonstrated significant ACD reduction accompanied by increases in LT, LV, and LAF, supporting progressive anterior segment crowding with aging. Importantly, age-stratified analyses suggested differences in the contributors to ACD shallowing. Among participants aged 50 to 60 years, follow-up to baseline changes in both LAF and LV were significantly associated with ACD reduction. LAF represents the relative size of the lens within the eye; a higher LAF indicates greater lens thickness relative to axial length, thereby reducing the anterior chamber space, contributing to ACD shallowing.^17^ LV represents the anterior position of the lens relative to the scleral spur; an increase in LV may result from an increase in lens size, anterior displacement of the lens, or a combination of both. In participants aged 50 to 60 years, both follow-up to baseline changes in LAF and LV were independently associated with ACD reduction, suggesting that ACD shallowing in this age group may be driven by both lens enlargement and anterior lens displacement.

In contrast, among participants over 60 years of age, ACD reduction was no longer associated with follow-up to baseline changes in LAF but remained significantly associated with follow-up to baseline change in LV. This suggests that in older individuals, continued relative lens enlargement may no longer be the main contributor to ACD shallowing. Under this condition, the remaining association between increased LV and ACD reduction may more likely reflect anterior displacement of the lens. The persistent increase in LV observed across age groups may be attributable to age-related zonular laxity or localized zonular dehiscence, which permits anterior movement of the lens–iris diaphragm and consequently results in a shallower central ACD.^18^ In addition, age-related changes in accommodative function may also influence anterior segment configuration, particularly in hyperopic eyes. Under the classic accommodative model, ciliary muscle contraction is associated with reduced zonular tension and lens thickening.^19^^,^^20^ Conversely, reduced accommodative drive with age may be expected to maintain relatively greater zonular tension and a flatter lens profile, potentially affecting anterior segment biometry.^21^ Although accommodative function was not directly assessed in this study, this mechanism warrants further exploration.

Taken together, our findings indicated that both lens thickening and anterior lens displacement contribute to ACD shallowing in earlier stages of aging. However, lens thickening is unlikely to continue indefinitely and may plateau beyond a certain age. Once lens thickening stabilizes, progressive anterior displacement, potentially driven by zonular laxity, may become the predominant mechanism underlying further ACD shallowing. These findings suggest that monitoring longitudinal changes in LAF and LV may provide additional context for understanding age-related anterior segment changes in PACS patients.

Previous studies have underscored the critical role of the lens in ACD reduction and angle closure.^16^^,^^22^ Praveen et al.^22^ reported a significant decrease in ACD with increasing lens thickness, while Chen et al.^23^ reported that LAF tended to increase progressively from normal eyes to PAC to PACG. Nongpiur et al.^24^ found that eyes with angle closure exhibited greater LV compared with normal eyes, Tan et al.^25^ demonstrated that LV was independently associated with narrow angles, and Ozaki et al.^26^ identified increased LV as a significant risk factor for angle closure. Consistent with these findings, our results showed that both lens thickening and lens forward movement with aging contribute to the progressive ACD reduction over a 5-year follow-up.

This study has several limitations. First, the selection of 60 years as a reference age was based on the median age of our study population and may require further validation in independent cohorts. With the expansion of health care access and advancements in cataract surgeries, an increasing number of participants are undergoing cataract surgery at a younger age, which limits the representation of older individuals. Second, this study included only individuals diagnosed with PACS and NPACS. The absence of participants with PAC or PACG limits our ability to evaluate the full spectrum of disease progression. Lastly, only Chinese participants were included, which may limit the generalizability of our findings to other populations.

## Conclusions

Our study showed that both PACS and NPACS eyes exhibit progressive shallowing of ACD over time. Age-stratified analyses suggested that factors associated with ACD reduction may differ by age, with both LAF and LV associated with ACD shallowing in relatively younger participants, while LV remained the main associated factor in relatively older participants.

## Supplementary Material

Supplement 1
