# Supplementary material for: Factors Associated With Anterior Chamber Depth Changes in Primary Angle-Closure Suspects Over 5-Year Follow-Up
Source: Transl Vis Sci Technol. 2026 Oct 23;15(10):37. doi: 10.1167/tvst.15.10.37 (PMC13715931; doi:10.1167/tvst.15.10.37)
Supplement: Supplementary file 1 [file tvst-15-10-37_s001.docx]

Table S1. Comparison of Baseline Demographic and Ocular Characteristics Between Two Age Groups

| Parameter | Baseline Age 50-60 years (N=128) | Baseline Age >60 years (N=109) | P value |
| --- | --- | --- | --- |
| Demographic Data |  |  |  |
| Gender (Male/Female) | 24/104 | 34/75 | 0.026‡ |
| Random blood glucose (IQR), mmol/L | 7.65 (5.93, 9.60) | 7.30 (6.00, 8.98) | 0.58§ |
| Heart rate (Mean ± SD), BPM | 84.32±12.16 | 82.56±12.17 | 0.27† |
| Ocular Data |  |  |  |
| BCVA (IQR) | 1.00 (0.80, 1.20) | 0.80 (0.80, 1.00) | **< 0.001**§ |
| IOP (IQR), mmHg | 17.00 (15.00, 19.00) | 16.00 (15.00, 18.00) | 0.06§ |
| SE (IQR), Diopter | 0.25 (-0.34, 0.88) | 0.88 (0.13, 1.50) | **< 0.001**§ |
| CCT (Mean ± SD), mm | 535.12±30.98 | 525.35±29.98 | 0.02† |
| LT (Mean ± SD), mm | 4.44±0.28 | 4.66±0.34 | **< 0.001**† |
| AL (Mean ± SD), mm | 22.81±0.83 | 22.86±0.80 | 0.64† |
| LAF (Mean ± SD) | 1.95±0.15 | 2.04±0.16 | **< 0.001**† |
| ACD (IQR), mm | 2.50 (2.30, 2.72) | 2.48 (2.22, 2.64) | 0.09§ |
| LV (Mean ± SD), mm | 0.46±0.26 | 0.54±0.25 | 0.02† |
| Iarea (Mean ± SD), mm2 | 1.42±0.17 | 1.42±0.18 | 0.43* |

PACS, primary angle closure suspect; NPACS, non-primary angle closure suspect; SBP, systolic blood pressure; DBP, diastolic blood pressure; BCVA, best corrected visual acuity; IOP, intraocular pressure; SE, spherical equivalence; CCT, central corneal thickness; LT, lens thickness; AL, axial length; LAF, LT/AL factor; ACD, anterior chamber depth; LV, lens vault; Iarea, iris area; SD, standard deviation; IR, interquartile range

†Two-sample t-test

‡ χ^2^ test

§Mann-Whitney test

*P value with adjustment for: pupil diameter

P-Values with statistical significance are shown in boldface.

Table S2. Comparison of Five-Year Changes of Anterior Chamber Parameters Between PACS and NPACS Participants

|  | NPACS (N=136) | PACS (N=101) | P value^a^ |
| --- | --- | --- | --- |
| △CCT,mm | -1.000(-5.000, 3.000) | 0.000 (-4.500, 4.000) | 0.17§ |
| △ACD, mm | -0.049(-0.097, -0.004) | -0.048 (-0.100, -0.011) | 0.40§ |
| △LT, mm | 0.130(0.070, 0.218) | 0.110 (0.070, 0.200) | 0.89§ |
| △AL, mm | 0.005(-0.030, 0.048) | 0.00(-0.030, 0.030) | 0.25§ |
| △LAF | 0.057(0.029, 0.089) | 0.054(0.031, 0.087) | 0.87§ |
| △LV, mm | 0.050(-0.035, 0.137) | 0.037(-0.023, 0.109) | 0.45§ |
| △I area, mm^2^ | 0.095(0.011, 0.171) | 0.083(0.015, 0.134) | 0.27* |

Δ, Follow-up-to-baseline changes (Follow up minus baseline); PACS, primary angle closure suspect; NPACS, non-primary angle closure suspect; CCT, central corneal thickness; LT, lens thickness; AL, axial length; LAF, LT/AL factor; ACD, anterior chamber depth; LV, lens vault; Iarea, iris area;

§Mann-Whitney test

*P value with adjustment for: pupil diameter

P-Values with statistical significance are shown in boldface

^a^ Bonferroni correction applied for multiple comparisons; significance threshold adjusted to α = 0.007 (0.05/7)
